# Silicon-Mediated Priming Induces Acclimation to Mild Water-Deficit Stress by Altering Physio-Biochemical Attributes in Wheat Plants

**DOI:** 10.3389/fpls.2021.625541

**Published:** 2021-02-19

**Authors:** Arruje Hameed, Tahir Farooq, Amjad Hameed, Munir Ahmad Sheikh

**Affiliations:** ^1^Department of Biochemistry, Government College University Faisalabad, Faisalabad, Pakistan; ^2^Department of Applied Chemistry, Government College University Faisalabad, Faisalabad, Pakistan; ^3^Nuclear Institute for Agriculture and Biology (NIAB), Faisalabad, Pakistan; ^4^Institute of Molecular Biology and Biotechnology (IMBB), The University of Lahore, Lahore, Pakistan

**Keywords:** seed priming, sustainable productivity, water-deficit stress tolerance, wheat, silicon

## Abstract

Water-deficit stress negatively affects seed germination, seedling development, and plant growth by disrupting cellular and metabolic functions, reducing the productivity and yield of field crops. In this study, sodium silicate (SS) has been employed as a seed priming agent for acclimation to mild water-deficit stress by invoking priming memory in wheat plants. In pot experiments, the SS-primed (20, 40, and 60 mM) and non-primed control seeds were allowed to grow under normal and mild water-deficit conditions. Subsequently, known methods were followed for physiological and biochemical studies using flag leaves of 98-day mature wheat plants. The antioxidant and hydrolytic enzymes were upregulated, while proteins, reducing sugars, total sugars, and glycine betaine increased significantly in the flag leaves of wheat plants originated from SS-treated seeds compared to the control under mild water-deficit stress. Significant decreases in the malondialdehyde (MDA) and proline contents suggested a controlled production of reactive oxygen species, which resulted in enhanced cell membrane stability. The SS priming induced a significant enhancement in yield, plant biomass, and 100-grain weight of wheat plants under water-deficit stress. The improvement in the yield parameters indicated the induction of Si-mediated stress acclimation in SS-primed seeds that elicited water-deficit tolerance until the maturity of plants, ensuring sustainable productivity of climate-smart plants.

## Introduction

Around the world, sustainable agriculture is facing severe threats from ecotoxicological conditions, climate change, and environmental stresses that pose serious challenges to global food security. Plants growing in a dynamic environment are heavily influenced by the aforesaid stress factors and exhibit a significant reduction in growth, development, and final yield, although they try to counterbalance the negative impacts through certain adaptive mechanisms, such as changing the osmotic potential, plant structure, and growth pattern, boosting the antioxidant defense potential and regulating physiological and biochemical processes (Teh and Koh, 2016). Over the years, drought or water deficit has been recognized as the most brutal environmental stress that retards the growth and development of plants by having negative impacts on the physiological, biochemical, and morphological traits. It hampers the normal metabolic, antioxidant, and photosynthetic activation and nutrient movement in plants. The disrupted processes at the subcellular level impair the growth-promoting parameters and lead to reduced plant growth and biomass and to yield losses (Wang et al., 2018; Easwar Rao and Viswanatha Chaitanya, 2020). Wheat is a major staple food, and its seed germination, seedling growth, and plant development also experience the drought-induced negative impacts, which ultimately result in low yield (Amirjani and Mahdiyeh, 2013; Guo et al., 2017). Wheat plants experience negative changes in protein contents, antioxidant potential, and hormone composition at the vegetative and reproductive stages under drought. It also influences the chlorophyll content, cuticle thickness, and opening and closing of the stomata (Bano et al., 2012; Guan et al., 2015; Li et al., 2020). In fact, water limitations severely reduce the uptake and translocation of macro- and micronutrients, which affect leaf–water relations, photosynthesis, and chlorophyll fluorescence, resulting in reduced plant growth, early senescence, and low wheat productivity (Zlatev, 2009; Karim et al., 2012; Wang et al., 2017).

Seed germination is one of the major phases in the life of higher plants in which all well-regulated metabolic, biochemical, and physiological processes ensure the rapid and uniform emergence of seedlings and plant development. Priming is a seed pre-conditioning technique that modulates the biochemical and physiological processes for the acceleration of germination and alleviation of stress, and for higher crop yields. In fact, it programs the seeds for the tolerance of abiotic stresses by regulating metabolism, antioxidant enzymes, and protein synthesis and readjusting the underlying subcellular pathways (Wojtyla et al., 2016; Hameed et al., 2019). Over the last few decades, a range of physical, chemical, and biological treatments have been well explored for hydropriming, chemopriming, biopriming, and thermoprining for pre-germinative metabolic modulations in seeds in order to withstand abiotic and biotic stress conditions at germination, seedling growth, and plant development. Various natural and synthetic priming agents (inorganic salts, organic molecules, and natural metabolites) have been reported to boost the antioxidant potential as a stress-responsive strategy for the alleviation of drought-induced damages in germinating seeds (Aranega-Bou et al., 2014; Savvides et al., 2016; Singh et al., 2020). Various studies have reported the applications of salicylic acid, abscisic acid, jasmonic acid, hydrogen peroxide, ascorbic acid, sodium nitroprusside, sodium chloride, sodium glutamate, etc., as wheat seed priming agents to induce tolerance against drought and to mitigate the above-mentioned negative impacts (Hameed and Iqbal, 2014; Bhardwaj et al., 2017; Habib et al., 2020).

Over the last few decades, several authors have reported the ability of silicon (Si) to induce tolerance against biotic and abiotic stresses, including salinity, high temperature, chilling, drought, etc. It accelerates seed germination and enhances plant growth and crop yield. It acts as a plant protectant and biostimulant under a range of stress conditions. It also improves the water status and water use efficiency of plants and reduces lipid peroxidation under drought stress (Hasanuzzaman et al., 2018; Liu et al., 2019). It could regulate osmolyte accumulation and readjust osmotic potential under water-deficit conditions. Si provision improved the yield of rice by increasing the mobilization of photoassimilates and amino acids from vegetative tissues to grain and nitrogen use efficiency. It redirected the primary metabolism by acting as a signaling factor under unstressed conditions (Pang et al., 2019; Mohanty et al., 2020).

Keeping the above facts in mind, this study was planned to employ Si as a wheat seed priming agent to induce acclimation to mild water-deficit stress until the maturity of plants.

## Materials and Methods

In this study, spring wheat (*Triticum aestivum* L. cv. AARI-2011) seeds (100 g for each treatment) were primed with 20, 40, and 60 mM sodium silicate (SS) solution, the Si donor, for 8 h. Then, they were washed and dried at 26 ± 2°C under shade. On the other hand, some wheat seeds were soaked only in water to achieve hydro-primed seeds for comparative study. Pot experiments with a completely randomized design were conducted in three replicates (five pots per replicate with five seeds per pot) to investigate the effects of silicon-induced priming on mature wheat plants produced from SS-primed seeds under normal and mild water-deficit conditions. Each pot was filled with 1.4 g cm^–3^ sandy clay loam soil containing 22% clay, 33% silt, and 45% sand. Normal growth conditions were maintained by providing water at soil water-holding capacity. On parallel, the drought stress was induced by maintaining water at 50% soil water-holding capacity. Physiological and biochemical analyses were performed using flag leaf samples collected from 98-day matured plants originated from SS-primed, hydro-primed, and non-primed control seeds grown under normal and mild drought conditions.

### Physiological Analyses

A known conductometric method was employed for the measurement of cell membrane stability (CMS) (Blum and Ebercon, 1981). The samples were autoclaved for 24 h before and after conductivity reading from the control (C) and stressed (T) leaf samples.

CMS % = [(1 − (*T*_1_/*T*_2_))/(1 − (*C*_1_/*C*_2_))] × 100

where *T*_1_ is the stress sample conductance before autoclaving, *T*_2_ is the stress sample conductance after autoclaving, *C*_1_ is the control sample conductance before autoclaving, and *C*_2_ control sample conductance after autoclaving.

The water status parameters (turgor, osmotic, and water potential) were measured using the youngest leaf samples according to known procedures. The difference between the water potential (*Ψ*_*w*_) and osmotic potential (*Ψ*_*s*_) values provided the leaf turgor potential (*Ψ*_*p*_).

(*Ψ*_*p*_ = *Ψ*_*w*_ − *Ψ*_*s*_)

### Biochemical Analyses

#### Biomolecules

Total soluble proteins (TSPs) were estimated by the method of Bradford using bovine serum albumin (BSA) as the standard (Bradford, 1976). Reducing sugars were determined using a well-known dinitrosalicylic acid method (Miller, 1959) and the total sugar content estimated using the phenol sulfuric acid reagent method (Dubois et al., 1951). The reducing and total sugar contents were determined from a standard curve prepared by using glucose as the standard. Non-reducing sugars were calculated by subtracting the reducing sugars from the total sugars.

#### Enzymatic and Non-enzymatic Antioxidants

For the different biochemical analyses, leaf samples (0.5 g) were ground in specific extraction buffer and centrifuged at 15,000 × *g* for 20 min at 4°C for different biomolecules and enzymes. Subsequently, the separated supernatant was employed for the below given biochemical analyses according to well-established methods. Spectroscopic analyses were performed using a spectrophotometer (Hitachi, U2800).

Specific extraction buffers were used to homogenize the leaf samples (0.5 g) as in a known procedure. The activity of superoxide dismutase (SOD) was assayed by measuring its ability to inhibit the photochemical reduction of nitroblue tetrazolium (NBT) following a known method (Giannopolitis and Ries, 1977). One unit of SOD activity was defined as the amount of enzyme which caused 50% inhibition of photochemical reduction of NBT. The activities of peroxidase (POD) and catalase (CAT) were also measured using a well-established method (Chance and Maehly, 1955). For POD activity, we noted an increase in the absorbance of the reaction solution at 470 nm. In the case of CAT activity, we recorded a decrease in the absorbance of the reaction solution at 240 nm. An absorbance variation of 0.01 U/min was taken as one unit of CAT and POD activities. Further, the activities of enzymes were expressed on seed weight basis.

The level of lipid peroxidation was measured in terms of malondialdehyde (MDA, a product of lipid peroxidation) content determined by a known method which involves thiobarbituric acid (TBA) reaction (Heath and Packer, 1968). An extinction coefficient of 155 mM^–1^ cm^–1^ was used for the calculation of MDA content. The Folin–Ciocalteu reagent was employed according to a known micro-colorimetric method for the determination of the total phenolic content (TPC) (Ainsworth and Gillespie, 2007). A linear regression equation was used for the measurement of TPC after preparing the standard curve with different concentrations of gallic acid.

#### Hydrolytic Enzymes

Protease activity was determined by a known casein digestion assay (Drapeau, 1976). The absorbance of the filtrate was measured at 280 nm. By this method, 1 U is the amount of enzyme that releases acid-soluble fragments equivalent to 0.001 *A*_280_ per minute at 37°C and pH 7.8. Enzyme activity was expressed on a protein basis. The α-amylase activity was determined by following a reported method (Varavinit et al., 2002). Accordingly, maltose was used for the construction of the standard curve to measure enzyme activity.

### Osmolytes

The known acid ninhydrin method was employed for the measurement of proline content at 520 nm and expressed as micrograms proline per gram fresh weight (FW) (Bates et al., 1973). A known spectroscopic method was adopted for the determination of glycine betaine (GB) (Grieve and Grattan, 1983). Warm distilled water was used to prepare an aqueous extract of dry leaves, which was mixed with 0.2 ml of potassium triiodide solution and 0.25 ml of 2 N HCl. After keeping this solution in an ice bath for 90 min, 20 ml of dichloromethane and 2 ml of water were added for the extraction of GB. The optical density of the organic phase was determined at 365 nm, and the GB concentrations were measured on a fresh weight basis.

### Pigment Contents

A known spectrophotometric-based method was followed to measure chlorophyll a (chl a), chlorophyll b (chl b), total chlorophyll (total chl), and carotenoids in leaf samples (Arnon, 1949; Peters and Noble, 2014). The pigments were extracted in 85% acetone and centrifuged at 4,000 rpm for 15 min; subsequently, the supernatant was used for the absorbance of the recorded values at 645, 663, and 480 nm. Afterward, the pigment contents were calculated as follows:

Chl⁢a⁢(mg/g⁢FW) =[12.7(OD)663-2.69(OD)645×V/1,000×W]

Chl⁢b⁢(mg/g⁢FW) =[22.9(OD)645-4.68(OD)663×V/1,000×W]

Carotenoids(mg/gFW)=[A/carEM]×1,000Acar =OD+4800.114(OD)663-0.638(OD)645

Where, OD represents optical density, *V* is the volume of the sample, *W* is the weight of fresh tissue taken for extraction, and EM is 2,500.

### Yield Attributes

The mature wheat plants were used to calculate the plant biomass, 100-grain weight yield, and the grain yield per plant under stress and non-stress conditions.

### Statistical Analyses

Finally, significance of the data was tested using variance and Tukey’s (honestly significant difference, HSD) test at *p* < 0.05 and, where applicable, at *p* < 0.01. Thus, the mean ± SD values are presented in graphs.

## Results

The SS-treated and non-treated wheat seeds were allowed to germinate in pots under normal and mild drought stress conditions. Later, the flag leaf samples of 98-day mature plants were used for biochemical, physiological, and yield responses and compared with the control under stress and non-stress conditions.

### Biochemical Parameters

**FIGURE 1:**
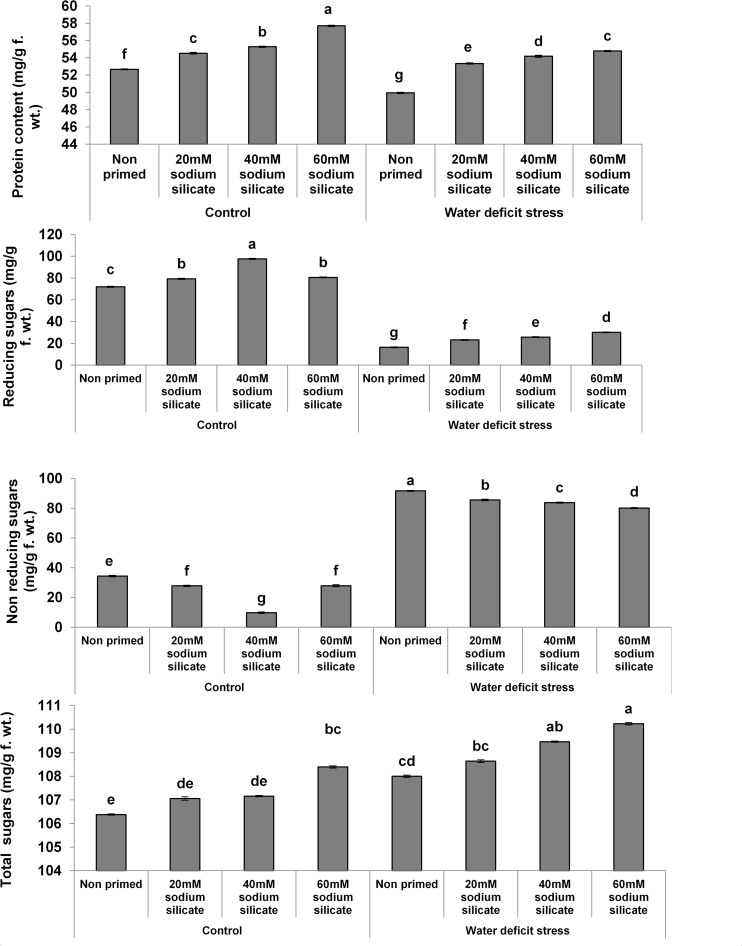
Contents of biomolecules in the flag leaf of wheat plants grown after sodium silicate seed priming under non-stress and water-deficit stress conditions. Each data point represents the mean of three samples ± SD. Bars with different alphabet are significantly different (*p* < 0.05 and *P* > 0.01) according to Tukey’s (HSD).

The protein contents increased significantly with increasing concentrations of the priming agent under normal as well as stress conditions compared to the control (Figure 1). There was a significant increase in reducing sugars with increasing concentrations of the applied sodium silicate priming solution under both conditions. However, the increasing effect was more pronounced in the flag leaves of plants originated from prime seeds under normal conditions. Interestingly, an entirely opposite effect was observed in the case of non-reducing sugars. The total sugars increased with increasing concentrations of the priming agent under drought, while under normal conditions, only priming with 60 mM SS treatment induced a significant increase in total sugars.

**FIGURE 2:**
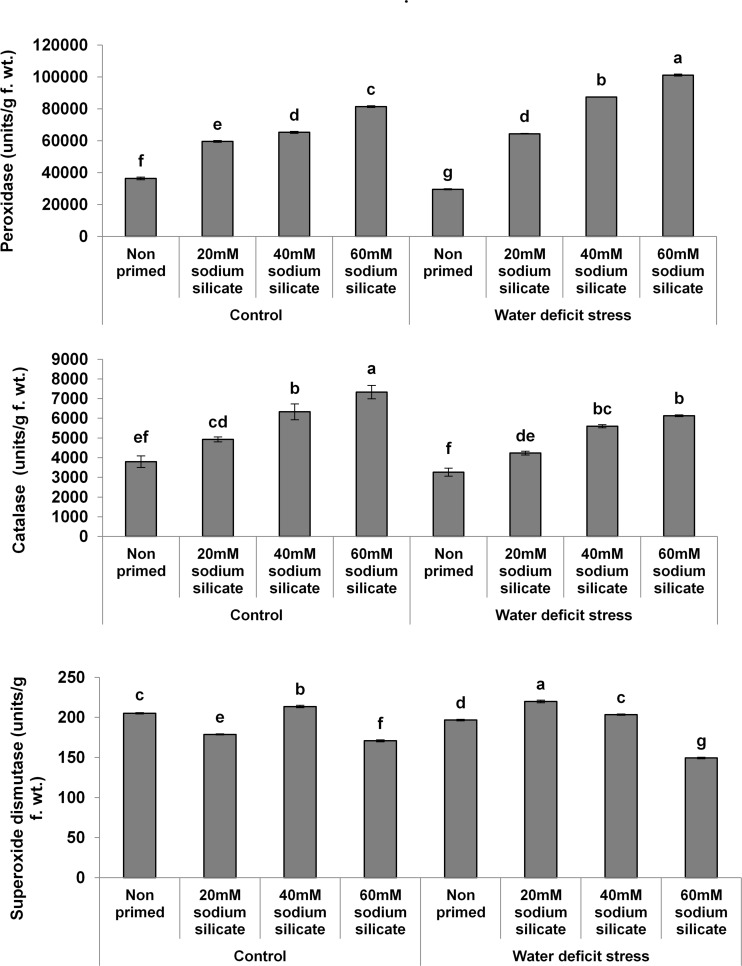
Antioxidant enzyme activities in the flag leaf of wheat plants grown after sodium silicate seed priming under non-stress and water-deficit stress conditions. Each data point represents the mean of three samples ± SD. Bars with different alphabet are significantly different (*p* < 0.05 and *P* > 0.01) according to Tukey’s (HSD).

In the case of the antioxidant enzymes, both POD and CAT activities were upregulated significantly with increasing priming concentrations under both conditions. However, a more pronounced effect was observed in POD and CAT under the mild drought and normal conditions, respectively. Priming with 20 and 40 mM SS induced a significant increase in SOD activity under drought. Under normal conditions, only 40 mM SS treatment caused a significant increase in SOD. The maximum upregulation of SOD was observed as a result of priming with 20 mM SS under stress (Figure 2).

**FIGURE 3:**
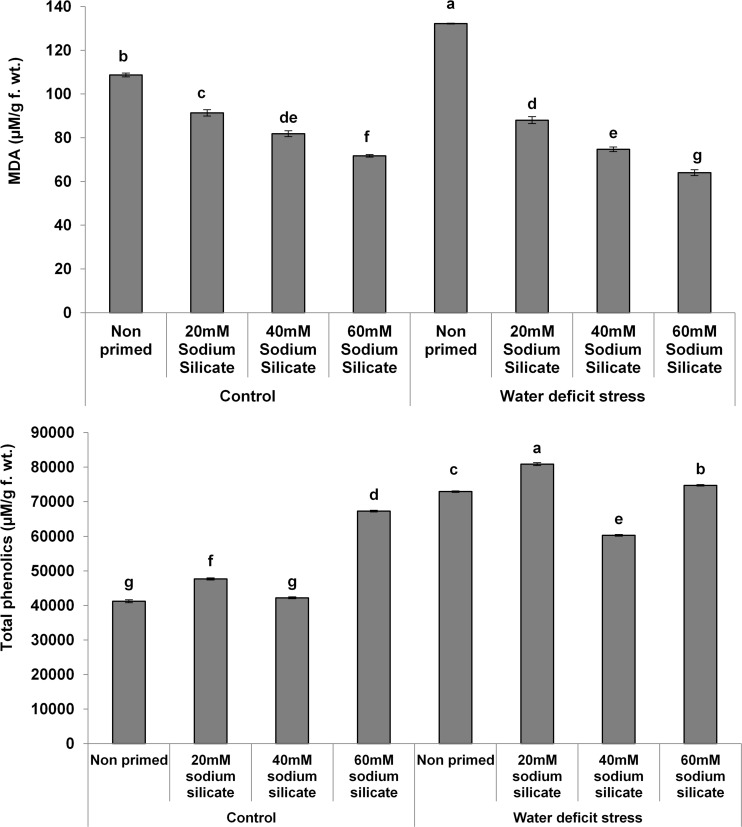
Malondialdehyde (MDA) contents and total phenolics in the flag leaf of wheat plants grown after sodium silicate seed priming under non-stress and water-deficit stress conditions. Each data point represents the mean of three samples ± SD. Bars with different alphabet are significantly different (*p* < 0.05 and *P* > 0.01) according to Tukey’s (HSD).

**FIGURE 4:**
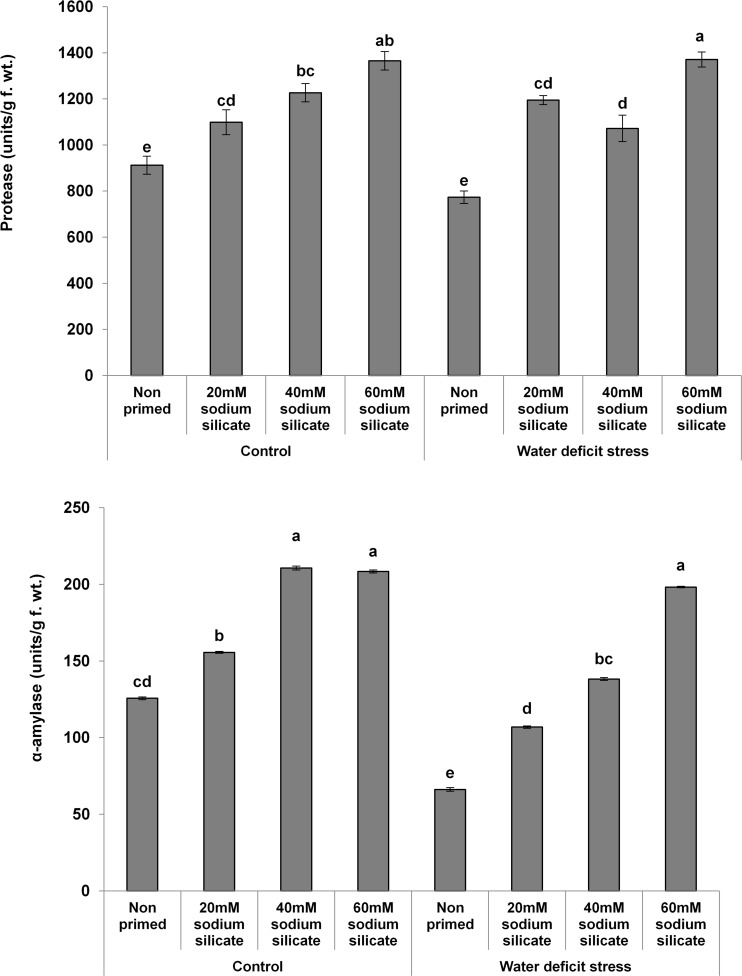
Hydrolytic enzyme activities in the flag leaf of wheat plants grown after sodium silicate seed priming under non-stress and water-deficit stress conditions. Each data point represents the mean of three samples ± SD. Bars with different alphabet are significantly different (*p* < 0.05 and *P* > 0.01) according to Tukey’s (HSD).

The MDA contents exhibited a significant decreasing trend with increasing priming concentrations. The maximum MDA was observed in the flag leaves of plants originated from non-primed seeds grown under drought. Treatment with 40 mM SS induced a significant decrease, while the other treatments caused an increment in the non-enzymatic antioxidant, the TPC, under both conditions compared to the control (Figure 3). In the case of the hydrolytic enzymes, the protease and α-amylase activities were significantly upregulated due to the priming treatments under normal as well as stress conditions (Figure 4).

**FIGURE 5:**
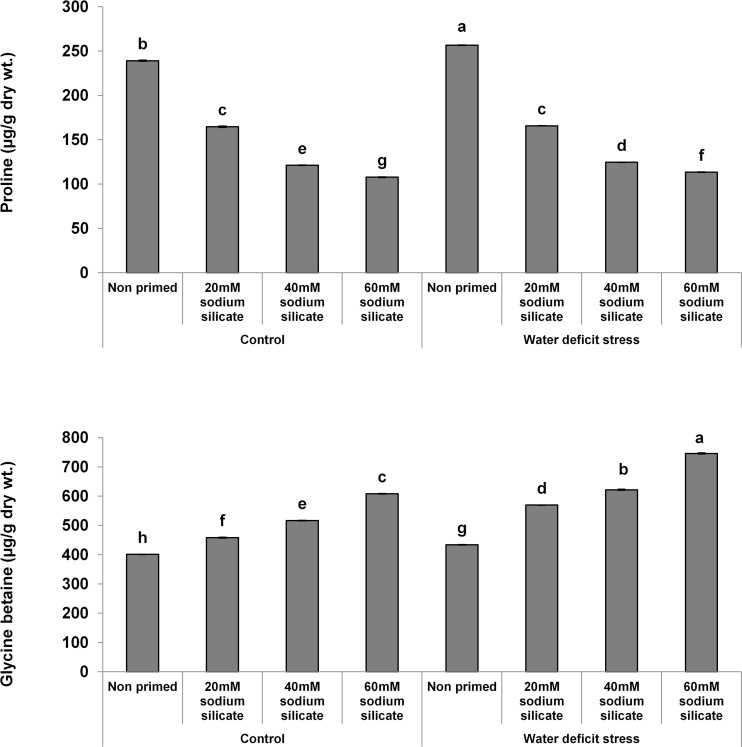
Osmolyte contents in the flag leaf of wheat plants grown after sodium silicate seed priming under non-stress and water-deficit stress conditions. Each data point represents the mean of three samples ± SD. Bars with different alphabet are significantly different (*p* < 0.05 and *P* > 0.01) according to Tukey’s (HSD).

The proline contents decreased significantly and linearly with increasing SS priming concentrations, while a significantly linear increase was observed in GB under both conditions (Figure 5).

Priming with 40 mM SS only caused a significant increase in chl a under normal conditions. The priming treatments induced a significant decrease in chl b and total chl under normal conditions. Under drought, priming with 20 and 60 mM SS caused a significant increase in chl a and chl b, respectively. Priming with 60 mM SS also increased the total chl significantly under stress. A significant increase in total carotenoids was observed as a result of priming with 20 and 40 mM SS under normal conditions. Meanwhile, priming with 60 mM induced a significant increase in total carotenoids under stress (Table 1).

**TABLE 1:** Effect of sodium silicate priming treatments on pigments.

Pigments	Treatments							
	
	Non-stress				Under water-deficit stress			
		
	Control	20 mM	40 mM	60 mM	Control	20 mM	40 mM	60 mM
		sodium silicate	sodium silicate	sodium silicate		sodium silicate	sodium silicate	sodium silicate
Chlorophyll a (μg/g FW)	335.611 ± 0.714d	334.837 ± 0.571d	356.237 ± 0.209a	335.090 ± 0.375d	335.274 ± 0.395d	342.317 ± 0.293b	330.613 ± 0.721e	339.277 ± 0.656c
Chlorophyll b (μg/g FW)	524.006 ± 0.827d	513.068 ± 0.855e	408.125 ± 0.730g	504.110 ± 0.628f	540.714 ± 0.781b	534.777 ± 0.311c	536.184 ± 0.522c	545.009 ± 0.575a
Total chlorophyll (μg/g FW)	859.617 ± 1.224d	847.905 ± 0.625e	764.362 ± 0.523g	839.200 ± 0.253f	875.988 ± 0.391b	877.094 ± 0.381b	866.796 ± 0.387c	884.287 ± 1.121a
Total carotenoids (mg/g FW)	32.529 ± 0.012e	32.783 ± 0.011d	32.995 ± 0.034c	32.207 ± 0.033f	33.129 ± 0.042b	32.516 ± 0.049e	33.180 ± 0.026ab	33.241 ± 0.017a

*FW, fresh weight. Mean values of three replicates are presented. Within a column, means sharing the same letters are non-significantly different (*p* > 0.05 and *p* > 0.01) according to Tukey’s HSD test.*

### Physiological Parameters

**FIGURE 6:**
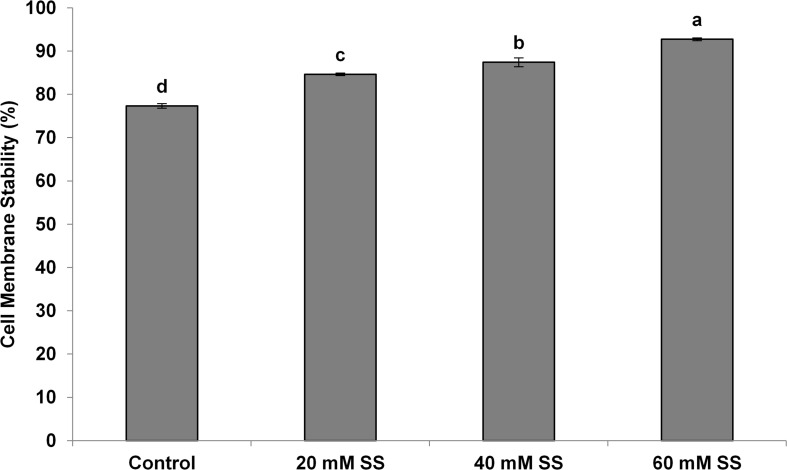
Effect of sodium silicate seed priming on cell membrane stability. Each data point represents the mean of three samples ±SD. Bars with different alphabet are significantly different (*p* < 0.05 and *P* > 0.01) according to Tukey’s (HSD).

There was a significant and linear decrease in the water potential of flag leaves with increasing SS priming concentrations under both conditions. The osmotic potential exhibited a similar trend under stress. However, a significant increasing effect was induced by 60 mM treatment under normal conditions. A significant increase in turgor potential was observed with increasing priming concentrations under both conditions (Table 2). CMS increased significantly with increasing SS priming concentrations (Figure 6).

**TABLE 2:** Effect of sodium silicate priming treatments on water status and yield parameters.

Water status/yield parameters	Treatments							
	
	Non-stress				Under water-deficit stress			
		
	Control	20 mM	40 mM	60 mM	Control	20 mM	40 mM	60 mM
		sodium silicate	sodium silicate	sodium silicate		sodium silicate	sodium silicate	sodium silicate
Water potential (MPa)	0.540 ± 0.008e	0.511 ± 0.002f	0.494 ± 0.002fg	0.475 ± 0.004g	0.967 ± 0.017a	0.875 ± 0.004b	0.705 ± 0.004c	0.566 ± 0.004d
Osmotic potential (MPa)	1.110 ± 0.002g	1.114 ± 0.002g	1.127 ± 0.001f	1.1338 ± 0.002e	1.343 ± 0.004a	1.295 ± 0.003b	1.218 ± 0.003c	1.176 ± 0.005d
Turgor potential (MPa)	0.570 ± 0.007d	0.603 ± 0.004c	0.633 ± 0.001b	0.664 ± 0.002a	0.376 ± 0.013g	0.420 ± 0.006f	0.513 ± 0.001e	0.611 ± 0.005c
Plant biomass (g)	4.011 ± 0.094e	5.111 ± 0.075c	5.833 ± 0.110ab	5,923 ± 0.019a	3.560 ± 0.143f	5.142 ± 0.048c	4.564 ± 0,030d	5.504 ± 0.171b
100-grain weight (g)	2.962 ± 0.028b	3.764 ± 0.104a	3.419 ± 0.084ab	3.286 ± 0.054ab	2.223 ± 0.096c	3.387 ± 0.106ab	2.819 ± 0.104bc	2.869 ± 0.104bc
Yield/plant (g)	1.217 ± 0.022ef	2.415 ± 0.068b	2.654 ± 0.098a	2.693 ± 0.033a	1.097 ± 0.023f	1.352 ± 0.003de	1.417 ± 0.011d	1.898 ± 0.089c

*Mean values of three replicates are presented. Within a column, means sharing the same letters are non-significantly different (*p* > 0.05 and *p* > 0.01) according to Tukey’s HSD test.*

### Yield Attributes

The yield of plants increased significantly with priming treatments under both conditions; however, the effect was more pronounced under stress-free conditions. A significant increase in 100-grain weight was observed only as a result of priming with 20 mM SS under normal and stress conditions, while the other treatments were unable to induce any perceptible change in it. The priming treatments induced a significant increase in plant biomass under both conditions (Table 2).

## Discussion

Applications of SS as a wheat seed priming agent induced a significant increase in TSP in the flag leaves of plants under both conditions. Proteins provide energy and amino acids to germinating seeds and developing seedlings and execute a number of physiological and biochemical processes in plants, being vital enzymes in various subcellular metabolic and signaling pathways. Drought stress induces severe negative impacts on such pathways due to their high dependence on water availability (Ali and Elozeiri, 2017). As a stress-mitigating strategy, plants produce drought-responsive proteins for biochemical readjustments to counterbalance the deleterious impacts and provision of resistance against stress (Al-Jebory, 2012). Silicon applications are known to enhance the expressions of drought-related proteins in rice plants under drought (Khattab et al., 2014). Accordingly, in our study, the Si-induced marked increment in TSP has been considered as a mechanistic response to water-deficit conditions. Hence, the significant improvement of the TSP content with increasing SS concentrations suggested the facilitating role of Si in protein synthesis under normal as well as stress conditions. The produced proteins could have assisted in the regulation of metabolic pathways and provided energy and nutrients for the induction of stress tolerance.

There were significant increases in the reducing and total sugars with increasing SS concentrations under stress and stress-free conditions. Plants increase their sugar content as a part of their stress-insulating mechanism because they could provide energy for metabolic processes and assist in the regulation of stress responses. Sugars do act as osmoprotectants and provide membrane stability, especially under water-deficit conditions (O’Hara et al., 2013; Poonam et al., 2016). Maize plants produced from Si-primed seeds were reported to show higher contents of soluble sugars under alkaline and water-deficit stress (Abdel Latef and Tran, 2016; Parveen et al., 2019). Hence, a Si-mediated increase in the reducing and total sugars has been considered as a positive factor for drought tolerance in this study. However, SS priming induced a significant decrease in non-reducing sugars, which suggested their lesser requirements in water-deficit management in this study.

In our case, the Si priming treatments significantly increased the POD and CAT activities in the flag leaves of wheat plants under normal and stress conditions. Priming with 20 and 40 mM SS caused a significant increase in SOD activity under stress and normal conditions, respectively, while all the other priming treatments induced a significant decrease in SOD under both conditions. In general, plants utilize non-enzymatic and enzymatic antioxidants for real-time detoxification of stress-induced reactive oxygen species (ROS). Therefore, upregulated antioxidant enzymes have been considered as a major stress-responsive strategy for counterbalancing excessive ROS (Sofo et al., 2015). The exogenous as well as priming applications of Si have been known to boost antioxidant enzymes in late-sown wheat and maize plants under drought (Sattar et al., 2017; Parveen et al., 2019). Thus, in our case, the elevated activities of the antioxidant enzymes represented an increased antioxidant potential for the provision of water-deficit tolerance in wheat plants.

The overexpression of the antioxidant enzymes along with the readjustment of biomolecules might have mitigated the negative impacts of water-deficit and helped to detoxify the excessive generation of ROS. It has been signified by the significant reduction of MDA contents with increasing priming concentrations prominently under mild water-deficit conditions. Under both conditions, two priming treatments induced a significant increase in TPC, a non-enzymatic antioxidant. The elevated antioxidant potential with reduced MDA contents suggested acclimation to water deficit in wheat plants induced as priming memory in primed seeds.

In our study, the activities of both hydrolytic (protease and α-amylase) enzymes increased significantly and almost linearly with increasing concentrations of SS under both conditions. Usually, plants show an upregulation of hydrolytic enzymes as a mechanistic approach for the mitigation of abiotic stresses including water deficit (Wang et al., 2016). They re-mobilize stored proteins and soluble sugars for the regulation of various metabolic and physiological functions vital for seed germination, seedling development, and plant growth (Adda et al., 2014; Martinez et al., 2019). In this perspective, the upregulation of the hydrolytic enzymes has been considered as a positive factor to elicit water-deficit tolerance in wheat plants.

Proline accumulation appears as one of the major stress-combating strategies in plants against a number of abiotic stresses, including water-deficit conditions. It helps the plants in osmotic readjustments for the development of water-deficit resistance. Further, it has been suggested as the biomarker of osmotic stress injury (Heuer, 2010; Chun et al., 2018). In our case, the proline contents were decreased significantly in the flag leaf of wheat plants with increasing priming concentrations compared to the control under both conditions. Accordingly, it represented that SS priming provided water-deficit acclimation in wheat plants, thus reducing the chances of osmotic injury and the requirement of proline. Glycine betaine is also produced in plants as an adaptive mechanism to counter abiotic stresses, including drought or water-deficit conditions. It is a known osmolyte that helps in stress-related osmotic readjustments for buffering of the redox potential, membrane stability, and higher ROS scavenging capacity. It interacts with the hydrophobic and hydrophilic domains of protein complexes and membranes, protects them from ROS, and maintains their structural and functional integrity. Thus, it offers counteracting mechanisms to settle the stress-related metabolic dysfunctions, ensuring improved growth and survival of plants (Xu et al., 2018; Annunziata et al., 2019). Silicon applications have regulated the osmolytes as a mechanistic approach to induce drought tolerance in different species of plants (Yang et al., 2017; Hasanuzzaman et al., 2018). In our case, there was a significant and linear increase in GB with increasing SS priming concentrations compared to the control under water-deficit and normal conditions. Therefore, the SS-mediated increase of GB in flag leaves has been suggested as a positive factor that elicits water-deficit tolerance in wheat plants.

Water-deficit conditions generally induce negative impacts on the chlorophyll content, resulting in a reduced photosynthetic activity (Chen et al., 2016; Hussain et al., 2019). In our case, SS priming produced significant increases in chl a, chl b, and total chl in flag leaves, suggesting the stress-mitigating role of Si under mild water-deficit conditions. Further, treatment with 60 mM SS caused a significant increase in total carotenoids under stress. In general, drought caused a significant reduction in carotenoid content in plants. They have been suggested as important precursors of plant hormones and accessory pigments for photosynthesis (Mibei et al., 2017). Foliar applications of Si also increased the photosynthetic pigments, including carotenoids, in wheat under normal and drought conditions (Maghsoudi et al., 2016). In a very recent study, silicon fertilization improved chlorophyll and carotenoids in sugarcane cultivars grown under water-deficit conditions (De Camargo et al., 2019). Thus, in our study, the silicon-mediated increase in photosynthetic pigments and carotenoids has been correlated to its stress alleviatory role for sustainable productivity.

In our case, the priming treatments resisted the negative impacts of mild water-deficit conditions and exhibited a significant increase in CMS in flag leaves. The increase in CMS has also been justified by a low MDA, a higher antioxidant potential, and higher amounts of osmolytes owing to the Si-regulated metabolic, biochemical, and signaling processes under mild water-deficit conditions. The increased CMS and the higher content of osmolytes developed osmoregulation, which significantly improved the relative water contents in flag leaves. Exogenous applications of Si have also been known to improve the CMS and water status in a variety of plants under different stress conditions (Bybordi, 2015; Maghsoudi et al., 2016; Altuntas et al., 2018).

All SS priming treatments induced biochemical alterations, consequently improving yield, plant biomass, and the 100-grain weight of wheat plants grown under mild water-deficit and normal conditions. The improvement of the yield parameters at the whole plant stage indicates the induction of priming memory in SS-primed seeds, which elicited water-deficit tolerance until the maturity of plants. Therefore, silicon-mediated priming memory in primed seeds enabled them to tolerate water deficit at the seed germination, seedling growth, and plant development stages, thus ensuring sustainable productivity with long-lasting stress acclimation.

## Conclusion

The silicon-mediated priming effects elicited water-deficit resistance in wheat plants by upregulating the antioxidant and hydrolytic enzymes with an increased level of osmoprotectants. Further, it induced a significant reduction in proline and MDA contents, representing a lesser ROS production that resulted in enhanced cell membrane stability. The improvement in the yield parameters at the whole plant stage indicated the induction of priming-mediated biochemical alterations in SS-primed seeds that invoked water-deficit acclimation until the maturity of plants, ensuring climate-smart growth of wheat.

## Data Availability Statement

The original contributions presented in the study are included in the article/Supplementary Material, further inquiries can be directed to the corresponding author/s.

## Author Contributions

ArH conducted the experiments and analysis of data, and performed the priming, growth, germination studies, and biochemical analyses. TF contributed to the data interpretation and manuscript writing. AmH supervised the priming, growth, germination studies, and biochemical analyses. MS proposed the research plan and supervised the priming, growth, germination studies, and biochemical analyses. All authors contributed to the article and approved the submitted version.

## Conflict of Interest

The authors declare that the research was conducted in the absence of any commercial or financial relationships that could be construed as a potential conflict of interest.
